# Long-term impact of liraglutide, a glucagon-like peptide-1 (GLP-1) analogue, on body weight and glycemic control in Japanese type 2 diabetes: an observational study

**DOI:** 10.1186/1758-5996-6-95

**Published:** 2014-09-08

**Authors:** Kana Inoue, Norikazu Maeda, Yuya Fujishima, Shiro Fukuda, Hirofumi Nagao, Masaya Yamaoka, Ayumu Hirata, Hitoshi Nishizawa, Tohru Funahashi, Iichiro Shimomura

**Affiliations:** Department of Metabolic Medicine, Graduate School of Medicine, Osaka University, 2-2-B5, Yamada-oka, Suita, Osaka, 565-0871 Japan; Department of Metabolism and Atherosclerosis, Graduate School of Medicine, Osaka University, 2-2-B5, Yamada-oka, Suita, Osaka, 565-0871 Japan

**Keywords:** Liraglutide, Glucagon-like peptide-1, Obesity, Diabetes, Metabolic syndrome, Eating behavior

## Abstract

**Background:**

Liraglutide, a glucagon-like peptide-1 (GLP-1) analogue, has been shown to possess pleiotropic effects including body weight reduction. However, long-term effect of liraglutide on body weight and glycemic control has not been elucidated in Japanese type 2 diabetes (T2D) subjects. Present study investigates whether liraglutide treatment maintains the body weight-decreasing and glucose-lowering effects for 2 years in Japanese T2D subjects.

**Methods:**

The enrolled subjects were 86 T2D patients [age; 59.8 ± 12.8 years, duration of diabetes; 15.8 ± 9.5 years, glycated hemoglobin (HbA1c); 8.5 ± 1.5%, body mass index (BMI); 27.3 ± 5.4 kg/m^2^ (15.8 - 46.5 kg/m^2^), mean ± SD]. Among 86 subjects, liraglutide was introduced in 25 inpatients and 61 outpatients, and 46 subjects were followed for 2 years. Clinical parameters were measured at baseline and 3, 6, 9, 12, and 24 months after liraglutide introduction. The increase in liraglutide dosage and the additional usage of glucose-lowering agents depended on each attending physician.

**Results:**

At 1 year after liraglutide introduction, 69 patients (80.2%) decreased body weight and 58 patients (67.4%) improved glycemic control. Body mass index (BMI) was changed 27.3 ± 5.4 kg/m^2^ to 25.9 ± 4.8 kg/m^2^ and percent reduction of body weight was significant and maintained over 4% at 2 years after liraglutide introduction. HbA1c was significantly decreased from 8.5 ± 1.5% to 7.7 ± 1.2% for 2 years. Liraglutide treatment tended to ameliorate lipid profile and hepatic enzymes. Stepwise regression analysis demonstrated that baseline BMI and previous insulin dose were positively associated with body weight reduction and baseline HbA1c was positively associated with reduction of HbA1c at 2 years after liraglutide introduction.

**Conclusions:**

Long-term liraglutide treatment effectively maintained the reduction of body weight and the fair glycemic control, and also improved lipid profile and liver enzymes in Japanese T2D subjects.

## Introduction

Recently, systematic analysis for the global prevalence of overweight and obesity has reported that the proportion of overweight adults gradually increased between 1980 and 2013 from 28.8% to 36.9% in male and from 29.8% to 38.0% in female [1]. The prevalence of type 2 diabetes has also increased worldwide [2] and similar increase was observed in Asia including Japan [3]. Evidently, obesity, especially visceral fat obesity, is located upstream of type 2 diabetes, hypertension, dyslipidemia, and atherosclerosis [4]. Obese type 2 diabetes subjects are more liable to cardiovascular diseases. Indisputably, the effective and efficient therapeutic strategy for obese type 2 diabetes should be developed and promoted. It is clear that the weight reduction is a therapeutic basis for obese type 2 diabetes. However, the management for obese type 2 diabetes often encounters difficulty in the control of appetite and body weight. In addition, treatment with insulin, sulfonylurea, and thiazolidinedione sometimes increase adiposity and such anti-diabetic treatments incidentally result in poor glycemic control because of weight gain.

Glucagon-like peptide-1 (GLP-1) is an incretin hormone with a potent blood-glucose lowering action only during hyperglycemia by inducing insulin secretion and reducing glucagon secretion in a glucose-dependent manner [5]. Beyond glucose-lowering effect, GLP-1 delays gastric emptying and induces satiety, leading to weight reduction. The mechanism is partly explained by the combination effects of GLP-1 on the gastrointestinal tract and the brain [6]. Native GLP-1 is immediately degraded and its elimination half-life is 1–2 min, whereas liraglutide has a long half-life, around 13 hours, and can be administered once a day [7]. A series of Liraglutide Effect and Action in Diabetes (LEAD) study showed the significant effect of liraglutide on weight reduction as well as glycemic control mainly in Caucasian diabetes subjects. Recently, long-term efficacy of liraglutide on weight reduction has been demonstrated in the sub-analyses of LEAD study [8, 9]. Our group has indicted the beneficial effects of liraglutide on visceral fat adiposity, body weight, and eating behavior until 6 months after liraglutide initiation in Japanese obese type 2 diabetes subjects [10, 11]. However, it has not been elucidated in Japanese type 2 diabetes subjects whether the effect of liraglutide on body weight and glycemic control would be maintained for longer period.

In Japanese type 2 diabetes subjects, we herein investigated the effects of liraglutide on body weight and glycemic control, and analyzed the association of clinical parameters and changes in body weight and HbA1c for 2 years after liraglutide introduction.

## Materials and methods

### Subjects

Present study was an observational study and the inclusion criteria were the subjects who were type 2 diabetes, introduced with liraglutide therapy at Osaka University Hospital (Osaka, Japan) between November 2010 and December 2012, and continued liraglutide treatment over one year. Number of subjects who were initiated with liraglutide during the indicated period was 136 diabetes patients. Among 136 patients, 22 patients returned to their home doctor, and thus 114 patients were followed up by our hospital. The 28 patients discontinued the liraglutide treatment within one year because of the following reasons: 11 patients suffered complications (digestive symptoms, severe loss of appetite, headache, and vertigo), 2 patients significantly improved glycemic control and discontinued liraglutide treatment, 14 patients deteriorated glycemic control and changed liraglutide treatment to other anti-diabetic agents, and 1 patient was diagnosed colon cancer. We finally analyzed 86 subjects who continued liraglutide treatment over one year, and 46 subjects were followed for 2 years among them. Among 86 subjects, 25 patients were introduced liraglutide during hospitalization and 61 patients were initiated liraglutide as an ambulatory treatment.

Physical examination and various metabolic parameters were measured at baseline and 3, 6, 9, 12, and 24 months after liraglutide introduction. Briefly, waist circumference was measured at the umbilical level in the late expiration phase at standing position by using a non-stretchable tape. Blood pressure was examined by using an aneroid sphygmomanometer at sitting position under resting condition in the consultation room. The increase in liraglutide dosage (0.3 mg/day, 0.6 mg/day or 0.9 mg/day, representing the maximum dose used in Japan) and the additional usage of glucose-lowering agents was depended and decided by each attending physician. The study protocol was approved by the human ethics committee of Osaka University and was registered with the University hospital Medical Information Network (Number: UMIN 000004192), and the informed consent was obtained from study subjects.

### Questionnaire for eating behavior

Eating behavior was assessed in part of patients before liraglutide treatment by using the questionnaire of The Guideline For Obesity issued by the Japan Society for the Study of Obesity. As reported previously [10], this questionnaire consists of 55-item questions of seven major scales as follows: 1) Recognition for weight and constitution (e.g.,‘Do you think it is easier for you to gain weight than others?’), 2) External eating behavior (e.g., ‘If food smells and looks good, do you eat more than usual?’), 3) Emotional eating behavior (e.g., ‘Do you have the desire to eat when you are irritated?’), 4) Sense of hunger (e.g. ‘Do you get irritated when you feel hungry?’), 5) Eating style (e.g., ‘Do you eat fast?’), 6) Food preference (e.g., ‘Do you like meat?’), 7) Regularity of eating habits (e.g., ‘Is your dinner time too late at night?’). All items were rated on a four-point scale ranging from 1 (seldom) to 4 (very often).

### Statistical analysis

Data of two groups were compared by the Student’s *t*-test or the Mann–Whitney test. The frequencies were compared between two groups by the *χ*^2^ test. The correlations between body weight, HbA1c and other parameters were first analyzed by simple regression analysis and then by multivariate stepwise analysis. In all cases, p values <0.05 were considered statistically significant. All analyses were performed with the JMP Statistical Discovery Software 8.0 (SAS Institute, Cary, NC).

## Results

### Characteristics of participants

Table 1 shows the baseline characteristics of 86 subjects before liraglutide introduction. The mean age and body mass index (BMI) were 59.8 years and 27.3 kg/m^2^, respectively, and BMI ranged from 15.8 to 46.5 kg/m^2^. Fifty-nine subjects (69%) were obesity in Japanese criteria (BMI ≥ 25 kg/m^2^). The duration of diabetes was 15.8 years and the mean hemoglobin A1c (HbA1c) was 8.5%. Eighty-six percent of patients had hypertension and 90% of patients had dyslipidemia. At baseline, 56% of patients were treated with insulin (average dosage: 26.9 units/day, maximum dosage: 93 units/day). These parameters indicate that the enrolled patients had relatively long duration of diabetes, relatively high frequency of obese subjects, high frequency of insulin use, and high dosage of insulin.

**Table 1:** **Baseline characteristics**

Sex (Male/Female)	44/42
Age (years)	59.8 ± 12.8
Duration of diabetes (years)	15.8 ± 9.5
HbA1c (%)	8.5 ± 1.5
Body mass index (kg/m^2^)	27.3 ± 5.4
Waist circumference (cm)	100.2 ± 11.3
LDL-C (mg/dL)	108.9 ± 33.4
HDL-C (mg/dL)	47.2 ± 13.8
Triglycerides (mg/dL)	136.9 ± 76.3
Hypertension (%)	86
Dyslipidemia (%)	90
Medication for Diabetes	
BG (%)	49
SU (%)	43
αGI (%)	22
TZD (%)	13
DPP4i (%)	17
Glinide (%)	3
Insulin (%)	56

Data are mean ± SD or number of subjects. LDL-C; low-density lipoprotein-cholesterol, HDL-C; high-density lipoprotein-cholesterol, BG; biguanide, SU; Sulfonylurea, αGI; α-glucosidase inhibitor, TZD; thiazolidinedione, DPP4i; dipeptidyl peptidase-4 inhibitor.

Patients were introduced with liraglutide treatment from 0.3 mg/day and its dosages were gradually increased according to glycemic control level by attending physicains. The number of patients treated with liraglutide at 0.3, 0.6, and 0.9 mg/day was 7, 24, and 55 patients at 1 year after liraglutide introduction, respectively, and such number of patients was 2, 8, and 36 patients at 2 year after liraglutide introduction, respectively.

### Changes of body weight and HbA1c at 1 year after liraglutide introduction

**Figure 1:**
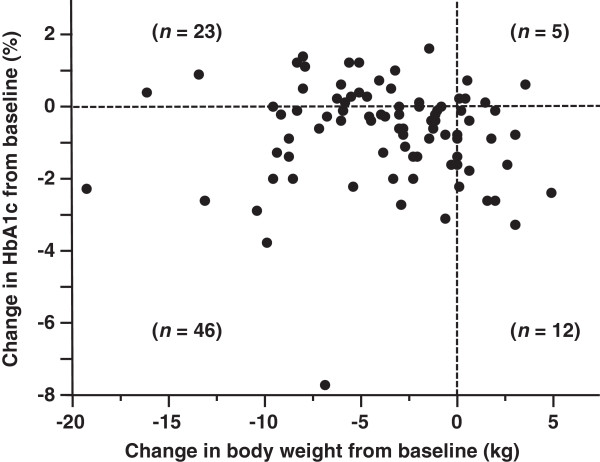
**Scatter plot of changes in body weight and HbA1c from baseline to 1 year after liraglutide introduction.**

In Figure 1, the individual changes in body weight and HbA1c from baseline to 1 year are shown as a scatter plot. The number of patients who reduced body weight was 69 cases (80.2%), and that of who improve glycemic control was 58 patients (67.4%). Forty-six patients (53.5%) were located in the lower left quadrant, i.e. both body weight and HbA1c were decreased in these patients.

Table 2 summarizes correlation analyses for the reduction of body weight or HbA1c from baseline to 1 year with baseline clinical parameters. Body weight reduction was associated positively with baseline BMI, aspartate aminotransferase (AST), alanine aminotransferase (ALT), baseline insulin dose, and previous insulin use. Stepwise regression analysis demonstrated that baseline BMI (*P* = 0.007) and previous insulin use (*P* = 0.027) were significantly and independently correlated with body weight reduction at 1 year. Reduction of HbA1c was associated positively with baseline HbA1c and negatively with insulin use for previous treatment. Lipids such as low-density lipoprotein-cholesterol (LDL-C) and triglyceride (TG) tended to be correlated with change in HbA1c. Stepwise regression analysis demonstrated that baseline HbA1c (*P* < 0.001) was correlated significantly and independently with the reduction of HbA1c.

**Table 2:** **Correlations between baseline parameters and the reduction of body weight or HbA1c at 1 year**

Baseline parameters	Reduction of body weight			Reduction of HbA1c		
	Univariate		Multivariate ***p***value	Univariate		Multivariate ***p***value
	*r*	*p* value		*r*	*p* value	
Age	0.006	0.955		−0.119	0.274	
Male	−0.129	0.236		−0.137	0.209	
BMI	0.355	0.001	0.007	0.082	0.456	
WC	0.210	0.114		−0.078	0.563	
Duration of DM	0.087	0.447		−0.072	0.533	
SBP	−0.158	0.146		0.002	0.986	
DBP	−0.050	0.649		0.135	0.216	
FPG	−0.066	0.549		−0.113	0.302	
HbA1c	−0.114	0.295		0.564	<0.001	<0.001
LDL-C	0.014	0.897		0.203	0.066	0.693
TG	−0.146	0.187		0.210	0.056	0.564
HDL-C	−0.014	0.902		−0.087	0.432	
AST	0.224	0.049		−0.128	0.265	
ALT	0.230	0.035	0.091	0.111	0.313	
sCPR	0.010	0.934		0.054	0.662	
Insulin dose	0.266	0.016	0.461	−0.057	0.611	
Insulin dose U/kg	0.213	0.055		−0.061	0.583	
Eating behavior	0.247	0.256		0.066	0.765	
Previous treatment						
Insulin	0.246	0.022	0.027	−0.225	0.037	0.374
BG	−0.063	0.562		0.050	0.645	
SU	−0.114	0.294		−0.046	0.284	
αGI	−0.046	0.672		0.117	0.284	
TZD	−0.072	0.503		0.054	0.622	
DPP4i	0.060	0.579		0.043	0.697	
Glinide	−0.032	0.770		0.046	0.676	

Reduction of body weight or HbA1c from baseline to 1 year was analyzed with baseline clinical parameters.

BMI; body mass index, WC; waist circumference, DM; diabetes mellitus, SBP; systolic body pressure; DBP diastolic body pressure, FPG; fasting plasma glucose, HbA1c; glycated hemoglobin, LDL-C; low-density lipoprotein-cholesterol, TG; triglycerides, HDL-C; high-density lipoprotein-cholesterol, AST; aspartate aminotransferase, ALT; alanine aminotransferase, sCPR; serum C-peptide immunoreactivity, BG; biguanide, SU; Sulfonylurea, αGI; α-glucosidase inhibitor, TZD; thiazolidinedione, DPP4i; dipeptidyl peptidase-4 inhibitor.

To exclude the effect of other anti-diabetic agents on body weight and glycemic control, we collected 35 patients whose anti-diabetic agents except liraglutide were not altered from baseline to 1 year (Table 3). Reduction of body weight was correlated significantly and positively with baseline BMI, ALT and the scores of the questionnaire for eating behavior, and was correlated negatively with systolic blood pressure. Stepwise regression analysis demonstrated that baseline BMI tended to be associated with body weight reduction. Reduction of HbA1c was correlated positively with baseline BMI, HbA1c, LDL-C, TG, and ALT, and negatively with baseline HDL-C and previous insulin use. However, there were no significant associations with the reduction of HbA1c and the baseline clinical parameters by stepwise regression analysis.

**Table 3:** **Baseline parameters and the reduction of body weight or HbA1c in the unchanged treatment group**

Baseline parameters	Reduction of body weight			Reduction of HbA1c		
	Univariate		Multivariate ***p***value	Univariate		Multivariate ***p***value
	*r*	*p* value		*r*	*p* value	
Age	−0.178	0.307		−0.147	0.401	
Male	−0.240	0.165		−0.053	0.764	
BMI	0.502	0.003	0.051	0.406	0.017	0.448
WC	0.294	0.208		0.243	0.302	
Duration of DM	−0.162	0.208		−0.052	0.784	
SBP	−0.370	0.029	0.243	0.011	0.949	
DBP	−0.111	0.527		0.238	0.169	
FPG	−0.107	0.540		−0.126	0.470	
HbA1c	0.165	0.344		0.863	<0.001	0.266
LDL-C	0.063	0.732		0.399	0.024	0.490
TG	−0.145	0.427		0.370	0.037	0.420
HDL-C	−0.035	0.850		−0.360	0.043	0.537
AST	0.331	0.069		0.218	0.240	
ALT	0.477	0.004	0.543	0.393	0.022	0.418
sCPR	0.050	0.822		0.256	0.239	
Insulin dose	0.105	0.555		0.023	0.897	
Insulin dose U/kg	0.015	0.933		−0.064	0.718	
Eating behavior	0.808	0.003	0.824	0.525	0.098	0.400
Previous treatment						
Insulin	0.214	0.217		−0.344	0.043	0.545
BG	0.017	0.921		−0.165	0.343	
SU	−0.190	0.273		−0.297	0.083	0.648
αGI	−0.054	0.757		−0.026	0.881	
TZD	−0.049	0.784		0.083	0.635	
DPP4i	0.031	0.861		−0.127	0.466	
Glinide	−0.036	0.836		−0.002	0.992	

Reduction of body weight or HbA1c from baseline to 1 year was analyzed with baseline clinical parameters in 35 subjects whose anti-diabetic agents were not altered except dosage of liraglutide.

BMI; body mass index, WC; waist circumference, DM; diabetes mellitus, SBP; systolic body pressure; DBP diastolic body pressure, FPG; fasting plasma glucose, HbA1c; glycated hemoglobin, LDL-C; low-density lipoprotein-cholesterol, TG; triglycerides, HDL-C; high-density lipoprotein-cholesterol, AST; aspartate aminotransferase, ALT; alanine aminotransferase, sCPR; serum C-peptide immunoreactivity, BG; biguanide, SU; Sulfonylurea, αGI; α-glucosidase inhibitor, TZD; thiazolidinedione, DPP4i; dipeptidyl peptidase-4 inhibitor.

### Changes in body weight, HbA1c, lipid profile and hepatic enzymes for 2 years

**Figure 2:**
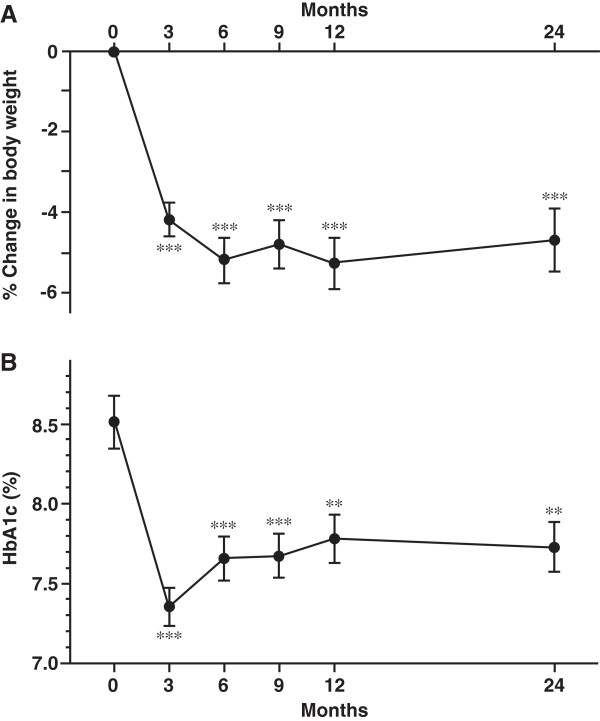
**Changes in body weight and HbA1c for 2 years after liraglutide introduction. A**, % Change of body weight. **B**, Change in HbA1c. Data are mean ± SE of 86 patients (0, 3, 6, 9, and 12 months) or 46 patients (24 months). **P < 0.01, ***P < 0.001, compared with the values at 0 month.

The 46 patients who continued to be treated with liraglutide over 2 years were next analyzed. BMI at 0, 3, 6, 9, 12, and 24 months were 27.3 ± 5.4, 26.3 ± 4.9, 25.8 ± 5.1, 25.9 ± 5.9, 25.8 ± 5.0, and 25.9 ± 4.8 kg/m^2^, respectively. Percent change in body weight indicated that body weight was significantly reduced and its reduction was maintained over 4% for 2 years (Figure 2A). HbA1c was significantly reduced at 3 months (7.4 ± 1.1%), and tended to increase at 6, 9, 12, and 24 months (7.7 ± 1.3%, 7.7 ± 1.3%, 7.8 ± 1.4%, and 7.7 ± 1.2%, respectively). Significant decrease of HbA1c was observed until 2 years after liraglutide introduction, compared to baseline HbA1c (Figure 2B).

**Figure 3:**
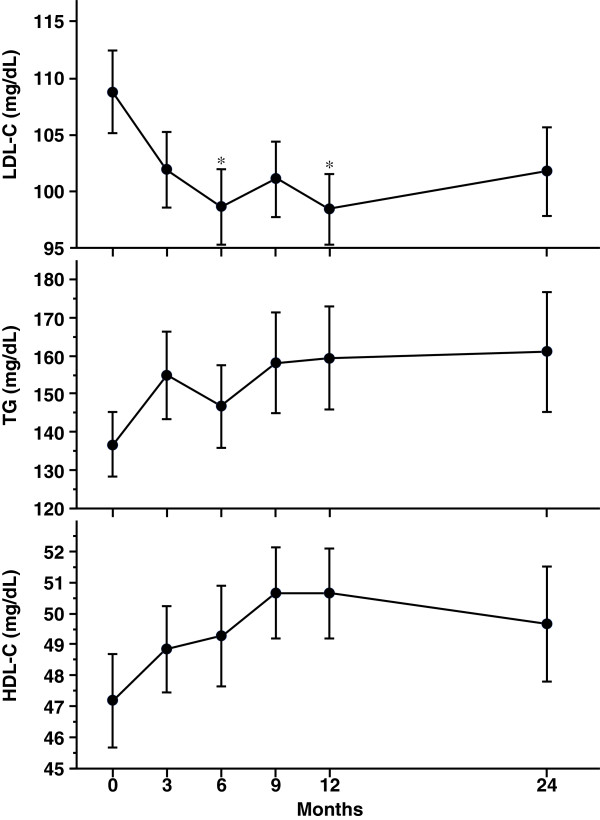
**Changes in lipids for 2 years after liraglutide introduction.** Data are mean ± SE of 86 patients (0, 3, 6, 9, and 12 months) or 46 patients (24 months). LDL-C, low-density lipoprotein-cholesterol; TG, triglyceride; HDL-C, high-density lipoprotein-cholesterol. *P < 0.05, compared with the values at 0 month.

**Figure 4:**
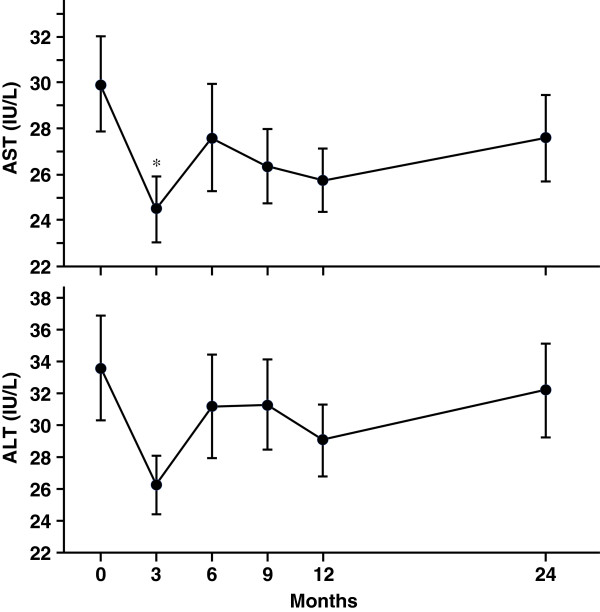
**Changes in liver enzymes for 2 years after liraglutide introduction.** Data are mean ± SE of 86 patients (0, 3, 6, 9, and 12 months) or 46 patients (24 months). AST, aspartate aminotransferase; ALT, alanine aminotransferase. *P < 0.05, compared with the values at 0 month.

Lipids (Figure 3) and hepatic enzymes (Figure 4) were also measured. LDL-C level was significantly reduced at 6 and 12 months, while high-density lipoprotein-cholesterol (HDL-C) tended to increase at 12 months (*P* = 0.098 versus 0 month). There were no significant changes of TG level from 0 to 24 months. AST level was significantly reduced at 3 months and tended to decrease at 12 months (*P* = 0.095 versus 0 month). ALT level also tended to reduce at 3 months after liraglutide introduction (*P* = 0.054 versus 0 month).

### Correlation of baseline clinical parameters with the reduction of body weight or HbA1c at 2 years

Table 4 summarizes correlation analyses for the reduction of body weight or HbA1c from baseline to 2 years with baseline clinical parameters. Reduction of body weight was positively correlated with baseline BMI, waist circumference (WC), and insulin dose. Stepwise regression analysis demonstrated that baseline BMI and insulin dose was significantly associated with body weight reduction for 2 years. Reduction of HbA1c was correlated positively with baseline HbA1c and LDL-C, and it was correlated negatively with sulfonylurea use for previous treatment. Stepwise regression analysis demonstrated that baseline HbA1c was significantly associated with the reduction of HbA1c.

**Table 4:** **Correlations between baseline parameters and the reduction of body weight or HbA1c at 2 year**

Baseline parameters	Reduction of body weight			Reduction of HbA1c		
			Multivariate ***p***value			Multivariate ***p***value
	*r*	*p* value		*r*	*p* value	
Age	−0.080	0.601		−0.197	0.190	
Male	−0.131	0.390		−0.052	0.732	
BMI	0.474	0.001	0.018	0.134	0.382	
WC	0.111	0.012	0.255	0.055	0.776	
Duration of DM	0.076	0.651		−0.003	0.984	
SBP	−0.106	0.490		0.003	0.982	
DBP	−0.007	0.963		0.222	0.138	
FPG	−0.115	0.451		−0.066	0.664	
HbA1c	0.145	0.341		0.738	<0.001	<0.001
LDL-C	0.137	0.383		0.420	0.005	0.701
TG	0.156	0.319		0.202	0.188	
HDL-C	0.024	0.880		−0.137	0.374	
AST	0.073	0.660		0.044	0.790	
ALT	0.002	0.988		0.232	0.120	
sCPR	0.222	0.152		0.108	0.542	
Insulin dose	0.345	0.023	0.027	−0.078	0.617	
Insulin dose U/kg	0.022	0.152		−0.102	0.509	
Eating behavior	−0.078	0.731		0.126	0.576	
Previous treatment						
Insulin	0.118	0.441		−0.176	0.242	
BG	−0.153	0.316		−0.053	0.725	
SU	−0.098	0.521		−0.250	0.094	0.996
αGI	−0.035	0.819		−0.006	0.969	
TZD	0.190	0.212		−0.191	0.204	
DPP4i	0.048	0.752		0.169	0.263	
Glinide	−0.060	0.697		0.161	0.285	

Reduction of body weight or HbA1c from baseline to 2 year was analyzed with baseline clinical parameters.

BMI; body mass index, WC; waist circumference, DM; diabetes mellitus, SBP; systolic body pressure; DBP diastolic body pressure, FPG; fasting plasma glucose, HbA1c; glycated hemoglobin, LDL-C; low-density lipoprotein-cholesterol, TG; triglycerides, HDL-C; high-density lipoprotein-cholesterol, AST; aspartate aminotransferase, ALT; alanine aminotransferase, sCPR; serum C-peptide immunoreactivity, BG; biguanide, SU; Sulfonylurea, αGI; α-glucosidase inhibitor, TZD; thiazolidinedione, DPP4i; dipeptidyl peptidase-4 inhibitor.

## Discussion

We demonstrated that liraglutide treatment decreased body weight and visceral fat adiposity, and ameliorated eating behavior until 6 months after liraglutide initiation in Japanese obese type 2 diabetes subjects [10, 11]. However, further long-term effect of body weight reduction and glycemic control by liraglutide has not been examined in Japanese type 2 diabetes subjects. Present study for the first time demonstrates the efficacy of liraglutide on weight reduction and fair glycemic control over 2 years in Japanese type 2 diabetes subjects. There are few reports describing the effect of liraglutide on body weight over 2 years. The LEAD-3 sub-analysis showed the significant weight reduction by liraglutide treatment for 2 years [8]. As shown in Figure 2A, liraglutide treatment for 2 years resulted in the 4.7% weight reduction (decrease of body weight: −3.4 kg from baseline). Present study suggests that over 4% of long-term body weight reduction can be expected by the low dose liraglutide treatment for Japanese patients, even with the additional other anti-diabetic agents causing weight gain such as insulin and sulfonylurea.

Evidently, the control of food intake cannot be ignored in the treatment of obese type 2 diabetes, but it is often difficult to control appetite and reduce adiposity in obese subjects. GLP-1 delays gastric emptying and induces satiety, which is probably related to the combined effect of GLP-1 on the gastrointestinal tract and the brain [12, 13]. Peripheral administration of liraglutide or lixisenatide can cross the blood brain barrier and enhanced cAMP level in brain, suggesting that GLP-1 receptor agonists directly acts on central nerve system [14]. Gastrointestinal and central nervous effect of GLP-1 receptor agonists synergistically exhibits the decrease of energy intake and body weight [15, 16]. Our previous studies demonstrated that liraglutide improved eating behavior in obese type 2 diabetes subjects until 6 months after liraglutide introduction [10, 11]. As shown in Table 3, reduction of body weight was correlated positively with the baseline scores of eating behavior in patients whose combined anti-diabetic agents were unchanged for 1 year. Present study suggests that liraglutide effectively reduces body weight especially in obese patients with the disordered eating behavior.

As in Figure 1, 46 patients (53.5%) resulted in the amelioration of both body weight and glycemic control. However, 23 patients (26.7%) revealed weight reduction but deterioration of glycemic control, while 12 patients (14.0%) showed improvement of glycemic control but weight gain. These results may imply that liraglutide-mediated ameliorations of body weight and glycemic control are independent and possibly by different mechanisms in GLP-1 actions. Fadini et al. recently reported in 166 patients (the average follow-up time: 9.4 months, the average daily dose of liraglutide at 16 months: 1.37 mg/day) that significant determinants of weight reduction or glycemic control were baseline BMI, or baseline HbA1c and previous insulin use, respectively [17]. Similar to their report, in the current study, significant determinants for the reduction of body weight or HbA1c from baseline to 2 year were baseline BMI and insulin dose, or baseline HbA1c, respectively (Table 4). Recent and current studies show that baseline BMI and HbA1c may be important predictors for weight reduction and glycemic control, respectively, before liraglutide therapy. Aiming at body weight reduction, especially in obese subjects with insulin therapy, the switch to liraglutide treatment may be more effective and beneficial in clinical use.

Several pleiotropic effects of GLP-1 receptor agonists have been demonstrated. GLP-1 receptor activation has revealed the cardiovascular protection [18, 19] and thus GLP-1 receptor agonist may be considered a promising new agent for the treatment of cardiovascular diseases linked to obese type 2 diabetes [20, 21]. GLP-1 receptor agonists have been shown to suppress glucagon secretion in mice and human [22]. Glucagon-mediated hyperglycemia may be attenuated by liraglutide treatment, but glucagon level was not monitored in present study. GLP-1 receptor agonists have been also shown to improve other cardio-metabolic parameters such as lipid profile and blood pressure following 5-10% weight reduction [23]. Similar to previous reports [23, 24], in present study, LDL-C was significantly reduced at 6 and 12 months even under statin treatment in 55% of patients. By using questionnaire for eating behavior, we previously showed the significant reduction of preference for fat at 6 months after liraglutide introduction, suggesting that the reduction of dietary fat intake partly contributed to the decrease of LDL-C level. Liraglutide treatment also showed the slight reduction of hepatic enzymes (Figure 4), which may indicate the improvement of fatty liver.

Present study has several limitations. This study is an observational study, not a randomized clinical trial (RCT) study. Dosage of liraglutide and combination with other glucose-lowering agents were depended on the attending physicians.

In summary, long-term treatment with liraglutide effectively reduced both body weight and HbA1c, and also improved lipid profile and liver enzymes in Japanese patients with type 2 diabetes. Significant independent determinants of weight reduction were baseline BMI and previous insulin therapy, suggesting that effect of liraglutide on weight reduction is further expected in obese type 2 diabetes patients with previous insulin therapy. Longer-term randomized clinical trials are warranted to more thoroughly elucidate the effect of liraglutide on body weight and complications of metabolic syndrome.
